# Editorial: The Role of Oxidative Stress, Epigenetics and Non-coding RNA in Regulating Trained Immunity

**DOI:** 10.3389/fimmu.2020.02114

**Published:** 2020-09-08

**Authors:** Mark W. Feinberg

**Affiliations:** Cardiovascular Division, Department of Medicine, Brigham and Women's Hospital and Harvard Medical School, Boston, MA, United States

**Keywords:** trained immunity, epigenetics, oxidative stress, non-coding RNA, stem cells

The adaptive immune response typically triggers antigen-specific memory T- and B-cells to protect against recurrent infections. However, accumulating studies demonstrate that innate immune cells, such as monocytes, macrophages, dendritic cells, and natural killer (NK) cells, are endowed with similar adaptive properties associated with immunological memory in a process that has been termed “trained immunity” (1). In support, studies performed in *Scid* or *Rag1-deficient* mice that harbor defective functional T- and B-cells, and consequently impaired adaptive immune responses, also show partial protection to reinfection (2). Bacteria, plants, and invertebrates, which also lack an adaptive immune response, also exhibit improved protection against reinfection highlighting the evolutionary conservation of this process (3). Moreover, after vaccination with live vaccines in human subjects, long-term functional changes of innate immune cells with enhanced antimicrobial function has been demonstrated. Finally, other traditionally considered “non-immune” cell types such as endothelial cells and vascular smooth muscle cells were shown to develop trained immunity when exposed to inflammatory challenges (4, 5).

While trained immunity of innate cells may confer additional level of protection against infectious pathogens, it may exacerbate chronic disease states, such as cardiovascular disease, in which heightened activation of innate cells generates a sustained and potentially uncontrolled inflammatory state that promotes disease pathogenesis and tissue injury. In this respect, trained immunity is a double-edge sword depending on disease context. This raises the question—what are the molecular mechanisms underlying the reprogramming of innate immune cells into cells with trained immunity? Emerging studies including those highlighted in this special issue indicate that long-term reprogramming depends on a combination of epigenetic and metabolic modifications.

As highlighted by Zhong et al., over-activation of the innate immune system in trained immunity has been proposed to contribute to the non-resolving inflammation in atherosclerosis. Furthermore, both infectious (e.g., lipopolysaccharide or β-glucan) and non-infectious stimuli [e.g., oxLDL, lipoprotein(a), aldosterone] are capable of priming innate cells such as monocytes to a trained phenotype upon re-challenge (6). Epigenetic remodeling at the level of histone methylation (e.g., H3K4me3) has been implicated in the development of trained immunity (6). Because monocytes exhibit a shorter lifespan than the duration of trained immunity, the long-term nature of trained immunity is likely elsewhere. Indeed, reprogrammed bone marrow progenitor cells, such as hematopoietic stem and progenitor cells (HSPCs) may serve as one reservoir for the generation of lineage-specific macrophages with endowed properties for trained immunity and sustained activation (7). In support of this hypothesis, 4 weeks after switching LDLR^−/−^ mice from a high cholesterol to a normal chow diet, monocytes from these mice surprisingly still develop a hyperactivated, pro-inflammatory state with augmented immune responses to TLR stimulation (8). Finally, human subjects with symptomatic atherosclerosis also harbor circulating monocytes with a trained pro-inflammatory phenotype (9).

Another important question addressed by Lai et al. is how do macrophage subsets develop phenotypic diversity and how does trained immunity contribute to specific subsets? Using microarray datasets of a range of tissue macrophages including 31 macrophage subset markers, 45 transcription factors in 34 diseases, including 10 types of cancers, and 23 trained immunity enzymes, they identify 12 shared and 20 group-specific disease macrophage pathways. They identify that peritoneum-derived and lung, liver, spleen, and intestine (LLSI)-derived macrophages express higher trained immunity enzyme transcripts than do ATM-derived macrophages. Collectively, interrogation of diverse macrophage datasets revealed new shared and divergent pathways of tissue macrophages and provides new hypotheses to limit inflammation and trained immunity in health and chronic disease.

The complex interplay of immune cell subsets is highlighted by Wagner et al. who focus on the harsh inflammatory microenvironment after myocardial infarction and why stem cell-based therapies may hold promise for controlling the immune response to facilitate myocardial repair. Ischemic injury is associated with myocardial necrosis and the elaboration of danger associated molecular patterns (DAMPs) and chemokines to recruit neutrophils and pro-inflammatory M1 macrophages that help clear necrotic tissues. In contrast, during the second phase, there is a shift to M2 pro-resolving, anti-inflammatory macrophages facilitated by CD4+ T regulatory cells. Other CD4+ T cells subsets are activated by autoantigens released from the injured myocardium. Stem cells, including mesenchymal stem cells release a range of paracrine factors, such as IL-10, TGF-β, and PGE2, that limit the immune response—likely a combination of adaptive, innate immunity, and trained immunity. Future studies will be required to further delineate the relative contribution of stem cell-based therapies on each of the arms of the immune response.

Another level of regulation of trained immunity is from the non-coding genome. Lin et al. highlight how long non-coding RNAs (lncRNAs) are regulated in response to infection by duck tembusu virus (STMUV), the causative agent of egg-drop syndrome in ducks. Because lncRNAs can interact with DNA, RNA, or chromatin, these findings provide new insights for potential compensatory feedback by viral infection. Future studies of re-challenging these cells with virus will be of interest to clarify specific lncRNAs in trained immune responses.

Finally, Carnino et al. highlight the potential roles of extracellular vesicles (EVs), their post-translational modifications and selective encapsulation of non-coding RNA cargo. Accumulating studies demonstrate that these EVs mediate intercellular communication involving the transfer of non-coding RNAs that exact functional and phenotypic alterations in recipient cells. The authors highlight multiple common types of post-translational modifications of EVs including protein deimination, a process generated by the peptidylarginine deiminase family of enzymes that converts arginine into citrulline and is implicated in the release of EVs. Interestingly, 42 essential metabolic and immune proteins are post-translationally deiminated in EVs only. Further investigation into the impact of these deiminated EVs and their non-coding RNA cargo on trained immunity will be of interest.

The collection of articles highlighted here aims to provide an overview of the current understanding of diverse facets of trained immunity and raise several important questions. Use of rigorous cell-based tools such as single-cell sequencing and epigenetic profiling tools such as single-cell ATAC-Seq may provide powerful insights for identifying specific cell types involved in trained immune cell responses and how they are regulated in disease-based contexts.

## Author Contributions

The author confirms being the sole contributor of this work and has approved it for publication.

## Conflict of Interest

The author declares that the research was conducted in the absence of any commercial or financial relationships that could be construed as a potential conflict of interest.
